# Knockout of Tmlhe in mice is not associated with autism spectrum disorder phenotypes or motor dysfunction despite low carnitine levels

**DOI:** 10.1186/s13229-023-00560-7

**Published:** 2023-08-08

**Authors:** Edgars Liepinsh, Baiba Svalbe, Gundega Stelfa, Solveiga Grinberga, Liga Zvejniece, Helgi B. Schiöth, Maija Dambrova

**Affiliations:** 1 Latvian Institute of Organic Synthesis, Riga, Latvia; 2 Latvia University of Life Sciences and Technologies, Jelgava, Latvia; 3 Uppsala University, Uppsala, Sweden; 4 Riga Stradins University, Riga, Latvia

**Keywords:** N6-trimethyllysine dioxygenase (TMLD), Autism spectrum disorder, Mice, Gamma-butyrobetaine, Mitochondria

## Abstract

Deletion of exon 2 of the trimethyllysine hydroxylase epsilon (*TMLHE*) gene was identified in probands with autism spectrum disorder (ASD). TMLHE encodes the first enzyme in carnitine biosynthesis, N6-trimethyllysine dioxygenase (TMLD). Researchers have suggested that carnitine depletion could be important for the development of ASD and cognitive, locomotor and social dysfunctions, but previous findings have been inconclusive regarding the specific role of endogenous carnitine. We developed a mouse knockout model with constitutive TMLD enzyme inactivation that exhibited a significant decrease in the carnitine by more than 90% compared to wild-type (WT) mice. However, we did not observe any significant social, cognitive, or repetitive-behavior changes associated with ASD in the knockout mice; muscle strength and coordination were also not affected. In addition, the life expectancy of knockout mice was similar to that of WT mice. In conclusion, knockout of Tmlh in mice does not induce an ASD phenotype or motor dysfunction despite extremely low carnitine and gamma-butyrobetaine concentrations. Moreover, inactivation of TMLD does not induce a phenotype similar to previously described primary carnitine deficiency; indeed, our results showed that low levels of carnitine sustained adequate energy production, muscle function and social behavior in mice.

**Supplementary Information:**

The online version contains supplementary material available at 10.1186/s13229-023-00560-7.

## Background

A search for genetic variants throughout the genome in individuals with autism spectrum disorder (ASD) and their families identified a novel deletion of exon 2 of the X-chromosomal trimethyllysine hydroxylase epsilon (*TMLHE*) gene [1]. Further studies identified such deletions in 16 probands with ASD, and these deletions are common in autistic but also healthy males [2]. This deletion results in loss of N6-trimethyllysine dioxygenase (TMLD) activity and the absence of a TMLD protein as well as metabolic abnormalities in plasma and urine [2]. *TMLHE* encodes the first enzyme in carnitine biosynthesis, TMLD, and enzyme deficiency was suggested to be a risk factor for ASD [2, 3]. Furthermore, this finding prompted speculation that carnitine metabolism is a target for therapeutic intervention [2]. Since mitochondrial dysfunction is associated with ASD [3], it was hypothesized that lower carnitine levels and changes in acylcarnitine profiles might be related to mitochondrial dysfunctions and abnormal fatty acid metabolism induced by deficiencies of carnitine synthesizing enzymes [2]. It has been speculated that this mechanism explains the many links of ASD with disturbed mitochondrial functions [4].

Carnitine is important for the mitochondrial metabolism of long- and medium-chain fatty acids. Endogenous carnitine biosynthesis begins with the TMLD-driven oxidation of TML and is then completed in three subsequent steps of enzyme-catalyzed reactions (5). The carnitine level in tissues is regulated by organic cation transporter novel type 2 (OCTN2, SLC22A5), which maintains carnitine homeostasis and ensures high tissue levels [6]. The main function of carnitine is the shuttling of long-chain fatty acid moieties across the inner mitochondrial membrane into the matrix for β-oxidation [6]. Therefore, carnitine supplementation is suggested for the treatment of several diseases related to insufficient mitochondrial activity [6].

In patients, primary carnitine deficiency is associated with inborn errors of carnitine biosynthesis enzymes, e.g., TMLD or a carnitine transporter. OCTN2 knockout (KO) mice are characterized by a 50% reduction in carnitine concentration, steatosis, hypoglycemia, intestinal ulcer formation, and decreased spontaneous activity, resulting in a 10% survival rate by one month after birth [7, 8]. However, in human studies of both children and adults, primary carnitine deficiency is characterized by highly variable clinical symptoms. The severity of symptoms varies from severe cardio-, hepato- and encephalopathy pathologies [6] to an asymptomatic course of disease in some individuals despite extremely low carnitine levels (i.e., 1–2 µM) in their plasma [9, 10]. These discrepancies might indicate that confounders are more important to the clinical manifestations of disorders than low carnitine levels per se.

To determine the impact of TMLD enzyme activity on the development of primary carnitine deficiency, we inactivated (knocked out) the Tmlhe gene (Tmlhe) in mice to study constitutive TMLD enzyme deficiency-induced changes in the levels of carnitine and its biosynthetic intermediates TML and γ-butyrobetaine (GBB) in plasma and brain tissue. In addition, we assessed ASD-related behavioral patterns of Tmlhe-KO and wild-type (WT) mice in a range of social behavior, cognitive and motor function tests.

## Materials and methods

A detailed description of methods is available in Additional file 1.

### Development of the Tmlhe-KO mouse model

The Tmlhe-KO mouse model was created using the CRISPR/Cas9 gene-editing protocol to generate random mutations [11]. Adult 11 weeks old C57BL/6 N mice (WT: 9 males and 14 females; Tmlhe-KO: 10 males and 11 females) were housed under standard conditions and tested for behavioral changes up till week 18. All WT (+/+) mice used in experiments were littermates of the KO mice. The experimental procedures were performed in accordance with the guidelines of the European Community and local laws and policies (Directive 2010/63/EU), and all of the procedures were approved by Food and Veterinary Service, Riga, Latvia.

### Levels of TML, GBB, and carnitine

TML, GBB and carnitine levels in plasma and brain tissue samples were measured by ultra-performance liquid chromatography-tandem mass spectrometry (UPLC‒MS/MS) in positive ion electrospray mode [5, 12].

### Behavioral tests to evaluate social behavior and motor function

We performed the commonly used tests to determine the mouse behavioral profile associated with ASD: three-chamber social, marble burying, and nest shredding tests [13]. Passive avoidance and Y-maze tests were used to determine cognitive function [14, 15]. Grip strength and rotarod tests were performed to assess muscle strength and coordination.

## Results and discussion

**Fig. 1:**
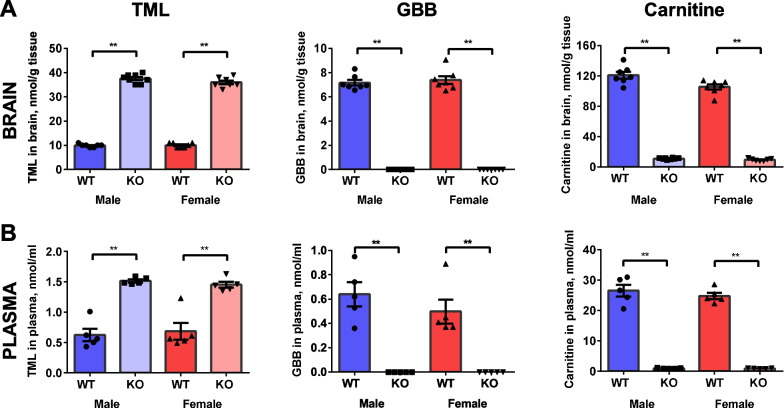
TML, GBB, and carnitine concentrations in WT and Tmlhe-KO mouse brains (**A**) and plasma (**B**). Concentrations of GBB and carnitine were significantly lower in KO mice than in WT mice. Each value is the mean (± SEM) of 5–8 mice. **p < 0.0001 compared to the WT group (two-way ANOVA followed by unpaired t-test). Coresponding F and p values are: plasma TML F_3, 16_ = 28.48, p < 0.0001; plasma GBB F_3, 16_ = 22.62, p < 0.0001; plasma carnitine F_3, 16_ = 168.6, p < 0.0001; brain TML F_3, 25_ = 741.5, p < 0.0001; brain GBB F_3, 24_ = 457.6; p < 0.0001, brain carnitine F_3, 25_ = 479.6; p < 0.0001

TML, GBB, and carnitine levels were substantially changed in both the plasma and brain tissues of Tmlhe-KO compared to those in their WT counterparts (Fig. 1), which is in line with the predicted functionality of the TMLD enzyme. We found up to 3.5-fold higher TML concentrations in plasma (1.51 ± 0.02 KO vs. 0.62 ± 0.10 WT nmol/ml) and brain tissue (37.50 ± 0.65 KO vs. 9.86 ± 0.26 WT nmol/g) of Tmlhe-KO mice than those of WT mice. GBB is an important intermediate metabolite in the carnitine synthesis pathway [16]. Since GBB was not detectable in Tmlhe-KO mouse plasma and brain tissues, we concluded that the carnitine synthesis pathway was severely interrupted in these animals. As a result, carnitine concentrations in Tmlhe-KO mouse plasma (0.92 ± 0.07 KO vs. 26.50 ± 1.93 WT nmol/l) and brain tissue (10.91 ± 0.39 KO vs. 121.1 ± 4.44 WT nmol/g) were significantly lowered to 90% of those in WT mice. Alterations in TML, GBB, and carnitine concentrations were observed in both male and female Tmlhe-KO mice. The Tmlhe-KO mice fully mimicked the most severe manifestations of inherited *TMLHE* gene deficiency in humans according to the carnitine, GBB, and TML levels.

We also compared the performance of the WT and Tmlhe-KO mice in a range of social behavior and motor function tests. In the three-chamber social test, Tmlhe-KO female and male mice displayed robust sociability by spending more time sniffing the novel mouse than the novel object (Table 1). In the marble-burying test, we observed that all mice were interested in marbles; there was no difference in marble burying between WT and Tmlhe-KO mice (Table 1). Nest-building behavior in the nest-building test also was unchanged in Tmlhe-KO mice (Table 1). Furthermore, Tmlhe-KO female mice demonstrated normal maternal behavior, including licking of pups, nest building, and crouching over grouped pups. We also tested mouse grip strength and performance on the accelerating rotarod test because both carnitine deficiency and ASD are associated with impairments in muscle strength and motor coordination [3]. The forelimb grip strength and rotarod performance of both male and female Tmlhe-KO mice was similar to WT mice, indicating that muscle strength and coordination in Tmlhe-KO mice were fully preserved despite very low carnitine concentrations (Table 1, Fig. 1). Patients with ASD might have variable degrees of intellectual disability [13]; therefore, we examined both working and contextual memory in mice using the Y-maze and passive-avoidance tests, respectively. We did not observe any differences in cognitive function between Tmlhe-KO mice and WT mice (Table 1). These results also confirmed that low levels of carnitine did not induce behavioral changes characteristic of ASD.

**Table 1:** WT and Tmlhe-KO mouse performance in social behavior, motor function and cognition tests

Tests	Male		Female	
	WT	Tmlhe-KO	WT	Tmlhe-KO
Social interaction (s)	88 ± 9	82 ± 14	79 ± 13	95 ± 20
Marble buried (%)	70 ± 12	86 ± 4	83 ± 6	80 ± 7
Nest build, score	8 ± 1	8 ± 1	6 ± 1	6 ± 1
Grip strength, g/g body weight	8 ± 0.3	8 ± 0.4	8 ± 0.6	8 ± 0.7
Rota-rod (s)	238 ± 20	242 ± 22	249 ± 16	233 ± 18
Passive avoidance (s)	86 ± 57	87 ± 33	100 ± 40	78 ± 26
Y-maze (% alternation)	60 ± 3	68 ± 5	69 ± 5	63 ± 5
Survival at 23 months of age (%)	55.6	63.6	75.0	81.8

Data are expressed as the mean ± SEM of 9–14 animals per group. Statistical analysis revealed no significant differences between Tmlhe-KO and WT mice in the performance of any behavioral test and survival (two-way ANOVA followed by unpaired t-test and Log-rank test to calculate Chi square of survival curves)

ASD has been associated with mitochondrial energy failure [3]. A retrospective study reported that 5% (vs. 0.01% in control) of children with ASD had a mitochondrial disease that was determined by abnormal concentrations of biomarkers such as increased plasma levels of lactate, alanine and acylcarnitines, which were suggested as markers of mitochondrial dysfunction [3]. The alanine plasma concentration was not increased in the Tmlhe-KO mice, but concentrations of lactate and acylcarnitines were significantly decreased, suggesting more efficient utilization of energy metabolism substrates in these mice [11]. Compared to WT mice, Tmlhe-KO mice exhibited improved mitochondrial functionality, particularly the OXPHOS-dependent respiration rate and OXPHOS coupling efficiency, in several tissues [11]. In a previous study, we reported that mitochondria in Tmlhe-KO mice are better protected against ischemia‒reperfusion-induced damage [11]. In contrast to OCTN2-deficient JVS mice [7, 8], signs of cardiac, muscle, and liver dysfunction were not observed over the 2-year lifespan of Tmlhe-KO mice, similar to WT mice (Table 1) [11]. This suggests that the transporter functions of carnitine or other organic cations might be more important for mitochondrial functionality than low levels of carnitine alone.

### Limitations

Despite the robust findings in the Tmlhe-KO mouse model, patients can be more sensitive to very low carnitine levels.

## Conclusions

In conclusion, constitutive Tmlhe gene inactivation in mice did not induce an ASD phenotype or motor dysfunction despite extremely low carnitine and GBB concentrations. The typical manifestations of primary carnitine deficiency were not induced by TMLD dysfunction, and low levels of carnitine did not result in impaired motor functions or any obvious ASD-like behavior.

### Supplementary Information

**Additional file 1:** A detailed description of methods and statistical analysis.

**Additional file 2:** Raw data of carnitine, GBB and TML measurements.

## Data Availability

All data generated or analyzed during this study are included in this published article and additional files (see Additional file 2). Any additional information related to the current study is available from the corresponding author on reasonable request.

## Abbreviations

ASD: Autism spectrum disorder
TMLHE: Trimethyllysine hydroxylase epsilon gene
TMLD: N6-trimethyllysine dioxygenase
WT: Wild-type
Tmlhe-KO: Trimethyllysine hydroxylase epsilon gene knockout mice
OCTN2, SLC22A5: Organic cation transporter novel type 2
GBB: γ-Butyrobetaine
UPLC‒MS/MS: Ultra-performance liquid chromatography-tandem mass spectrometry
OXPHOS: Oxidative phosphorylation
JVS: Juvenile visceral steatosis
