# Supplementary material for: Knockout of Tmlhe in mice is not associated with autism spectrum disorder phenotypes or motor dysfunction despite low carnitine levels
Source: Mol Autism. 2023 Aug 8;14:29. doi: 10.1186/s13229-023-00560-7 (PMC10410785; doi:10.1186/s13229-023-00560-7)
Supplement: Supplementary file 1 — Additional file 1: A detailed description of methods and statistical analysis. [file 13229_2023_560_MOESM1_ESM.docx]

**Supplementary Materials**

**Materials and Methods**

## Content of animal R70 chow (Lantmännen Lantbruk, Sweden)

Ingrediients: Oat flour, rye grain, wheat bran, flour, beet fiber, potato protein, minerals, lysine, vitamins and trace elements.

| Energy, % | | |  | **Vitamins** (supplemented) | |
| --- | --- | --- | --- | --- | --- |
| Metabolizable energy | 1254 kJ/100g | |  | A | 9000 IU /kg |
| Total protein | 14.5 | |  | D | 1000 IU/kg |
| Total fat | 4,5 | |  | E | 50 mg/kg |
| Linoleic acid | 1.0 | |  | K3 | 15 mg/kg |
| N free extracts | 60.1 | |  | B1 | 6 mg/kg |
| Total fibers | 4.9 | |  | B2 | 5 mg/kg |
| Ash | 5.0 | |  | B6 | 5 mg/kg |
| Water | <11 | |  | B12 | 0.07 mg/kg |
| Minerals, % | | |  | Biotin | 0.4 mg/kg |
| Ca | 0.7 | |  | Folic acid | 1.0 mg/kg |
| P | 0.5 | |  | Calcium pantothenate | 15 mg/kg |
| NaCl | 0.4 | |  | Niacin | 20 mg/kg |
| Mg | 0.25 | |  |  |  |
| K | 0.8 | |  |  |  |
| Microelements, mg/kg | | |  |  |  |
| Cu | | 12 |  |  |  |
| Fe | | 145 |  |  |  |
| Mn | | 68 |  |  |  |
| Zn | | 80 |  |  |  |
| I | | 1,3 |  |  |  |
| Co | | 0,5 |  |  |  |
| Se | | 0,1 |  |  |  |

## Development of the Tmlhe-KO mouse model

The Tmlhe-KO mouse model was created in the Laboratory Animal Centre of Tartu University using the CRISPR/Cas9 gene-editing protocol to generate random mutations. A chemically modified CRISPR Revolution EZ sgRNA kit (TMLHE2.1 AGUUGUUGCACCAACAAAGC, Synthego Corporation, USA) and CRISPR Evolution Cas9 2NLS nuclease protein (Synthego Corporation) were used to target exon 2 of the *TMLHE* gene. Embryo electroporation (of fertilized egg cells at 0.5 days postconception) was performed using an in-house validated protocol using a Nepagene Nepa21 electroporator and a CUY501P1-1.5 electrode. The following electroporation parameters were used: poring pulse (voltage = 50 V, pulse length = 2.5 ms, interval = 50 ms, number of pulses = 4, decay rate = 10%, and polarity (+)) and transfer pulse (voltage = 5 V, pulse length = 50 ms, interval = 50 ms, number of pulses = 5, decay rate = 40%, and polarity (+/-)). The resulting founder lines were analyzed by pyrosequencing to detect random mutations in exon 2 of the *TMLHE* gene. The most successful founder Tmlhe-KO line had a deletion of 2 nucleotides, 103352 and 103353, located on the second coding exon in the *Tmlhe* gene in chromosome X (NT_165789 region: 251286.469175). Nucleotides missing in Tmlhe-KO animals resulted in a frameshift and multiple STOP codons after the first 13 amino acid coding triplets.

Adult 11weeks old C57BL/6 N mice (WT: 9 males and 14 females; Tmlhe-KO: 10 males and 11 females) were housed under standard conditions (21-23 °C, reversed 12:12-h light/dark cycle, relative humidity of 45-65%) with unlimited access to food (R70 diet from Lantmännen, Stockholm, Sweden) and water and and tested for behavioral changes up till week 18. Generation of the Tmlhe-KO mouse line was approved by the Estonian Project Authorization Committee for Animal Experiments (no. 103, 22nd of May 2017). Female mice both heterozygous and homozygous for the *Tmlhe* gene were crossed with WT male mice and KO male mice. All WT (+/+) mice used in experiments were littermates of the KO mice. The experimental procedures were performed in accordance with the guidelines of the European Community and local laws and policies (Directive 2010/63/EU), and all of the procedures were approved by Food and Veterinary Service, Riga, Latvia. All experiments were performed in a blinded manner.

Behavioral testing was performed by experienced testers blinded to the experimental groups. Behavioral tests were performed during the dark phase of the light/dark cycle under red illumination (lights off at 08:00 and lights on at 20:00). Only one behavioral test was performed daily, separately one for male mice and the other day for female mice, so the hormone fluctuations did not affect the behavior. Behavioral tests that cause less stress were performed first, and the most stressful tasks were done as the final, with sufficient intervals between testing days. Tests were used in following order: marble buried, grip strength, rota-rod, Y-maze, nest build, social interaction, and passive avoidance test.

***Determination of carnitine, GBB, and TML levels in plasma and brain tissue***

Simultaneous determination of carnitine, GBB, and TML levels in plasma and brain homogenates was performed by liquid chromatography-tandem mass spectrometry (LC/MS/MS) (1, 2). Analyte extraction was performed by sample deproteinization with an acetonitrile/methanol mixture (3:1, v/v). To improve the detection sensitivity for TML, it was derivatized with 6-aminoquinolyl-N-hydroxysuccinimidyl carbamate (AQC). Carnitine and GBB in the same sample remained underivatized, as AQC reacts only with primary and secondary amino groups. The internal standard (IS) 3-(2,2-dimethyl-2-prop-1-yl-hydrazinium)propionate was applied for calibration. Sample processing was as follows. First, 20 µL of plasma or brain tissue was deproteinized by the addition of 80 µL of ACN/MeOH (3:1, v/v), and the mixture was vortexed and centrifuged at 10,000 × g for 10 min; then, 10 µL of supernatant was mixed with 50 µL of borate buffer solution (0.1 M, pH 8.1) and 20 µL of reagent solution (3 mg/mL AQC in acetonitrile); next, samples were vortexed and incubated at 55 °C for 10 min to complete the derivatization reaction; afterward, samples were allowed to cool to room temperature and 700 µL of IS solution (5 ng/mL in ACN/MeOH, 3:1, v/v) was added; finally, 50 µL of the resulting sample was diluted with 450 µL of ACN/MeOH (3:1, v/v), and 1 µL of the final solution was injected into LC/MS/MS system.

LC/MS/MS was carried out using a Shimadzu LCMS-8060NX system. A Waters Acquity BEH Hilic 1.7 µm, 2.1 × 50 mm column was used. Chromatographic separation was performed in a 10 mM ammonium acetate (pH 4.0) and acetonitrile gradient at a flow rate of 0.25 mL/min. MS/MS data acquisition was performed in positive electrospray ionization using multiple reaction monitoring mode with the precursor-to-product ion transitions of m/z 162.0→102.9 for carnitine, m/z 146.0→87.1 for GBB, m/z 359.1→189.2 for TML AQC derivative, and m/z 175.1→58.1 for IS.

***Behavioral tests***

**A three-chamber social assay** was used to characterize sociability, preference for social novelty, and social recognition. The test was conducted as described previously (3). The apparatus was a rectangular three-chambered box (each chamber: 42 (width) × 20 (length) × 22 (height), in cm) made of clear polycarbonate. Retractable doorways built into the two dividing walls controlled access to the side chambers. Mice were moved to the room 1 h before testing. Testing was conducted in a dim, red-lit room. Testing consisted of three consecutive 10-min phases. The first phase consisted of a 10-min habituation period while confined to the center chamber, followed by a 10-min habituation period with access to all 3 empty chambers. The mouse was then briefly confined to the center chamber while a novel object (a stainless steel wire cup) was placed in one of the side chambers. A stimulus mouse (novel to the focal mouse and previously habituated to the enclosure) was placed in an identical wire cup located in the other side chamber. The side chambers that contained the novel object and the novel mouse alternated across subjects to eliminate side bias. After the novel object and the novel mouse were positioned, the two side doors were simultaneously lifted, and the focal mouse was allowed to explore all three chambers for 10 min. The chamber and cups were cleaned with 70% ethanol between each animal. Animals used as novel mice (age-, strain- and sex-matched) were habituated to sitting under the wire cup for 10 min over three consecutive days. If an animal showed aberrant behavior, such as bar biting, excessive self-grooming, circling, or clinging to the sidebars with all four paws after the third day, it was excluded. Interaction was measured by the time spent sniffing the novel object or the novel mouse using a stopwatch.

**The marble-burying assay** is a tool for assessing anxiety-like and/or repetitive behaviors in mice (3). Subjects were tested in a regular clean cage (48-cm long × 25-cm wide × 20-cm high) with 5 L of fresh bedding. The focal mouse was first placed in the empty test cage (without marbles) for a 10-min habituation period. Then, the mouse was temporarily moved to another empty clean cage, while 15 blue glass marbles were positioned over the bedding in the test cage in an equidistant, 3 × 5 arrangement that covered the bedding surface. The subject was then returned to the test cage and allowed to explore and bury the marbles during a 30-min session that was videotaped. At the end of the session, the subject was removed, and the number of marbles buried to at least two-thirds of the depth was counted.

**The nest-building** **test** is used to assess escape behavior and hyperreactivity in mice *(3)*. Nests are necessary for heat conservation and reproduction, serving as shelter for mice. Mice were temporarily housed in individual cages containing no environmental enrichment items (such as bedding, nesting material, cardboard houses or chew sticks) to test their ability to build nests. Thirty minutes before the dark phase, mice were placed in a clear home cage with two cotton wool packages (cotton wool PREMIUM Zig-Zag, Livsane). The test was repeated twice and scored on the morning following the second test using the following variables adapted from (3) (maximum score =11): nestlet shredding, scored as 0 (not at all), 1 (partially shredded), or 2 (fully shredded); nestlet dispersion, scored as 0 (nestlet dispersed all over the cage), 1 (mostly used to build a nest), or 2 (fully used to build a nest); nest density, scored as 0 (not dense), 1 (medium density), or 2 (high density); nest shape, scored as 0 (no nest), 1 (ball shape), 2 (nest shape but no bottom), or 3 (full nest); and the presence of walls, scored as 0 (no walls), 1 (partial wall), or 2 (fully surrounded by walls).

**A** **grip strength** **test** for mice was used to assess muscle strength. The animal was placed over a base plate (Grip Strength Test Meter for Mice, IITC Life Science, USA) in front of a grasping bar. The animal was held by its tail and was gently passed over the mesh until it grasped the grid with its paws. The grip strength was determined in grams. The mean value of three trials was calculated for each mouse. Between each trial, mice were given a 5-min rest. The mean grip strength was then normalized according to body weight.

**The rotarod test** (Model 47600; Ugo Basile) was used to measure motor coordination. Briefly, on the first day, mice were pretrained on the rotarod apparatus (5 rpm) with one 300-s session per animal. On the experimental day, mice were placed on the rotarod, which had an accelerating rotating speed (from 5 to 40 rpm), for 300 s with a 30-min rest between trials. The time spent walking on the accelerating rotarod before falling off was measured. The mean of three trials was calculated for each mouse.

**A passive avoidance test** was performed to assess learning and memory in mice as previously described (4). Briefly, on the training day, each mouse was individually placed in the light compartment of an apparatus with no access to the dark compartment and allowed to explore for 60 s (Ugo Basile, Comerio, Italy). After this time, the sliding door (4 × 4 cm) was automatically opened, and the mouse was allowed to crossover into the dark compartment. Upon entering the dark compartment, the mouse received a shock of 0.2 mA for 3 s and was enclosed in the dark compartment for 20 s before being returned to its home cage. A retention test was performed on the next day (24 h later) without any shock. The time before entering the dark compartment was recorded as the retention latency. The maximum retention latency was set at 540 s.

Working memory performance was assessed by recording spontaneous alternation behavior in a **Y-maze**, as previously described (5). The experiment was conducted in a dim, red-lit room. The mice were individually placed at the end of one arm in a symmetrical Y-shaped runway (arm length of 35 cm, arm width of 5 cm, wall height of 21 cm) and allowed to explore the maze for 5 min. An alternation was defined as consecutive entries into all three arms. The total number and sequence of the arm entries were manually recorded, and the percentage of alternation was calculated.

***Statistical analysis***

Data are expressed as mean ± SEM, and statistical comparisons were made using two-way ANOVA followed by unpaired *t*-test and survival rates of WT and Tmlhe-KO mice were estimated by Log-rank test. p < 0.05 was considered statistically significant. Statistical analysis was performed using GraphPad Prism 8.1 software (Graph Pad Prism software, USA).

**References**

1. A. Kazaks *et al.*, Expression and purification of active, stabilized trimethyllysine hydroxylase. *Protein Expr Purif* **104**, 1-6 (2014).

2. E. Liepinsh *et al.*, Inhibition of L-carnitine biosynthesis and transport by methyl-gamma-butyrobetaine decreases fatty acid oxidation and protects against myocardial infarction. *British journal of pharmacology* **172**, 1319-1332 (2015).

3. E. Drapeau, M. Riad, Y. Kajiwara, J. D. Buxbaum, Behavioral Phenotyping of an Improved Mouse Model of Phelan-McDermid Syndrome with a Complete Deletion of the Shank3 Gene. *eNeuro* **5** (2018).

4. L. Zvejniece *et al.*, Investigation into stereoselective pharmacological activity of phenotropil. *Basic Clin Pharmacol Toxicol* **109**, 407-412 (2011).

5. L. Zvejniece *et al.*, The cognition-enhancing activity of E1R, a novel positive allosteric modulator of sigma-1 receptors. *British journal of pharmacology* **171**, 761-771 (2014).
